# Detection of minimal residual disease in circulating cell-free DNA in acute myeloid leukemia

**DOI:** 10.1038/s41598-025-20589-3

**Published:** 2025-09-23

**Authors:** Charlotte Sommer, Hildegard I. D. Mack, Madeleine C. Killer, Petra Ross, Andrea Nist, Thorsten Stiewe, Andreas Neubauer, Cornelia Brendel, Elisabeth K. M. Mack

**Affiliations:** 1 Department of Hematology, Oncology and Immunology, Philipps-University Marburg, and University Hospital Gießen and Marburg, Campus Marburg, Marburg, Germany; 2 Genomics Core Facility, Institute of Molecular Oncology, Universities of Gießen and Marburg Lung Center, Member of the German Center for Lung Research (DZL), Philipps-University Marburg, Marburg, Germany; 3 Institute of Lung Health, University Gießen, Gießen, Germany; 4 Present Address: Department of Hematology, Medical Oncology and Palliative Medicine, St. Marien-Krankenhaus Siegen, Siegen, Germany

**Keywords:** Acute myeloid leukemia, Cell-free DNA, Minimal residual disease, Next-generation sequencing, Leukaemia, Next-generation sequencing

## Abstract

Minimal/measurable residual disease (MRD) in Acute Myeloid Leukemia (AML) is defined as persistent leukemic cells below cytomorphological detection threshold. Next generation sequencing (NGS) of circulating cell-free DNA (cfDNA) to profile cancer-associated mutations has been shown to allow for quantification of disease burden in solid tumors and has also been suggested to enable minimally invasive follow-up of AML patients. In this pilot study we investigated the technical sensitivity and potential prognostic implications of cfDNA-based MRD monitoring in AML after allogeneic stem cell transplantation in comparison to donor chimerism analysis or, respectively, after consolidation chemotherapy. 75 cfDNA samples from 29 patients were analyzed by targeted NGS using a commercially available 10- or 37-gene hotspot panel (VariantPlex Core AML or Core Myeloid panel, ArcherDx). Patients’ leukemias exhibited 1–7 mutations as determined by routine diagnostics. Only previously identified mutations were considered for MRD evaluation. cfDNA was isolated in sufficient amounts for NGS from all samples (total yield 24 ng–5.2 µg). The sensitivity of variant detection increased with higher overall read count and higher mutation-specific coverage (variant allele frequency [VAF] range 0.08–100%). At least one previously known mutation was identified in 32/55 samples (58%, VAF 0.08–78.04%) which were taken during hematological complete remission (CR) in both patients after allogeneic stem cell transplantation (aHSCT) and patients after consolidation chemotherapy. In patients after aHSCT (*n* = 25), at least one previously known mutation was detected in 16/29 cfDNA samples (55.1%, VAF 0.08–6.7%) obtained when donor chimerism was ≥ 90% and in 6/6 samples (100%, VAF: 0.88–63.77%) with reduced donor chimerism. Probability of progression-free survival 17 months after aHSCT in patients with donor chimerism ≥ 90% but mutation-positive cfDNA was 64% compared to 100% in patients with undetectable MRD. In patients after consolidation chemotherapy, cfDNA was positive in all samples taken during CR (*n* = 4; VAF 0.26–29.84%) and non-CR (*n* = 4; VAF 8.46–100%). Our results indicate that NGS of cfDNA is suitable for MRD monitoring in AML and offers higher sensitivity for detecting residual leukemic cells than chimerism analysis in patients after aHSCT. Further studies are needed to evaluate clinical relevance of MRD status as determined in cfDNA.

**Supplementary Information:**

The online version contains supplementary material available at 10.1038/s41598-025-20589-3.

## Introduction

Acute Myeloid Leukemia (AML) accounts for approximately 80% of acute leukemias in adults with about 4.2 in 100.000 people newly diagnosed per year. Overall prognosis for AML is poor with a relative five-year survival rate of 31,9%^1^. AML is routinely diagnosed based on cytology of peripheral blood or bone marrow, most importantly the detection of > 10–20% blasts, in combination with cytogenetics and molecular genetic analyses to detect specific AML-related mutations such as *NPM1*, *CEBPA*, *FLT3*-ITD/-TKD or *IDH1/2*. The precise pattern of genetic aberrations is key to risk stratification and therapy selection^2^. While patients carrying aberrations associated with favorable prognosis receive chemotherapy as consolidation therapy, patients with intermediate or poor prognosis receive allogenic hematopoietic stem cell transplantation (aHSCT), if a suitable donor is available, the patient’s performance status is sufficient and the patient agrees to this hard and potentially complicated procedure^2^.

Most patients display somatic AML-related mutations already at the time of initial diagnosis^3^. Monitoring AML-associated mutations during and after therapy complements cytology or flow-cytometry-mediated detection of residual blasts as a measure of minimal/measurable residual disease (MRD)^4^. In patients who have received aHSCT, donor chimerism is determined by PCR-based SNP genotyping to enable early detection of relapse^5^. MRD is defined as detectable disease below the sensitivity-threshold of 5% blasts that can be achieved by morphology-based examination^6^. Laboratory methods for MRD-monitoring include flow cytometry and molecular techniques such as PCR, qPCR and NGS and achieve sensitivities of 10^−4^ to 10^−6^^2,6^.

NGS offer the advantage that many genes can be analyzed for mutations in a single assay with a short turnaround time of less than five days by employing multigene panels relevant to AML. Thus, NGS-assays require less input material compared to multiple parallel (q)PCR-assays, while providing sufficient sensitivity for mutation detection - at least at initial diagnosis^7,8^. For both, PCR/qPCR- and NGS-based methods, DNA extracted from bone marrow (BM) or peripheral blood (PB) or circulating cell-free DNA (cfDNA) has been used as input.

cfDNA is released through active secretion or upon apoptotic or necrotic cell death and its levels are higher in cancer patients than in healthy controls^9,10^, as well as in later tumor stages and in metastatic settings^10–12^. cfDNA has been used successfully to monitor disease burden in both solid tumors and hematologic malignancies, including lymphomas, multiple myeloma and myelodysplastic syndrome^13–15^. More precisely, concentrations of cfDNA are typically < 25 ng/ml in healthy individuals, with > 80% originating from hematopoietic cells, while levels in cancer patients have been reported to be increased > 20-fold^16–18^. In contrast to BM, PB and cfDNA can be sampled rather gently for the patient by venous puncture. BM on the other hand is the recommended source for MRD-analysis from a technical point of view if maximum technical sensitivity is desired^19^. However, cfDNA has been shown to enable detection of leukemia-specific mutations or mixed chimerism in patients after aHSCT with at least equal sensitivity and in many cases even earlier than PBMC-derived DNA by digital PCR^20,21^.

In this pilot study, we used a straight-forward assay incorporating commercially available gene panels that had not been optimized for the analysis of cfDNA by the manufacturer to monitor mutational burden in cfDNA of AML patients by NGS. We investigated the technical sensitivity of the approach and potential prognostic implications after both aHSCT or, respectively, consolidation chemotherapy.

## Materials and methods

### Patients and samples

This pilot study was approved by the clinical ethics committee at the Faculty of Medicine, Philipps-University Marburg (ethical vote 137/16) and all patients provided written informed consent to participate. All patients included in this study were treated for AML at the University Hospital Marburg. Treatments were chosen appropriately according to patient age, performance state, and disease- and patient-specific factors. cfDNA blood samples were collected during routine controls during hospitalization and outpatient follow-up between February 2017 and June 2019, but there was no fixed schedule for sample collection. Collection of reference samples during follow-up was performed according to the clinical standard procedures of our department, including routine remission controls after therapy and a structured after-care post alloHSCT, or, respectively at time points were there was a clinical suspicion of relapse (Supplementary Table S1). A total of 75 samples from 29 patients was selected retrospectively for cfDNA-analysis by NGS based on the presence of AML-associated mutations at initial diagnosis as reported by the reference laboratory and the availability of samples relative to a relapse of the disease (if applicable) as determined by routine diagnostics. Reference laboratory results and patients’ clinical courses were extracted from electronic health records.

### Isolation of CfDNA

cfDNA was isolated from blood samples collected in cell-free DNA blood collection tubes (Streck, La Vista, NE, USA) using the QIAamp Circulating Nucleic Acid Kit (Qiagen, Hilden, Germany) according to the manufacturer’s instructions. cfDNA yield was determined with a Qubit 3.0 Fluorometer (Life Technologies, Darmstadt, Germany) and quality was assessed in a PreSeq DNA QC Assay (Archer Dx, Boulder, Boulder, CO, USA) on a CFX96-Real-Time-PCR-Detection-System (BioRad, Munich, Germany) as recommended.

### Sequencing library preparation

Sequencing libraries were prepared using the VariantPlex Core AML (10 genes) or Core Myeloid panels (37 genes) (ArcherDx, Boulder, CO, USA; cf. Supplementary Table S2 and S3). Libraries were quantified by qPCR using NEBNext-Library-Quant-Kit for Illumina (New England Biolabs, Ipswich, MA, USA) and a CFX96-Real-Time-PCR-Detection-System (Bio-Rad, Munich, Germany). 150 bp paired-end sequencing was performed on a MiSeq or NextSeq instrument (Illumina, San Diego, CA, USA). Flow cell loading was calculated so that the minimum read number recommended by the manufacturer for each panel could theoretically be obtained (Core AML: 0.75 × 10^6^ reads; Core Myeloid: 3 × 10^6^ reads).

### Bioinformatics analysis

Sequencing reads were analyzed using the Archer Analysis bioinformatics pipeline (v6.0) in single analysis mode for somatic mutation calling. Potential germline variants were not considered since our patient cohort did not include any individuals with known or suspected germline predisposition for AML. Moreover, germline variants are difficult to interpret in the setting of MRD in cfDNA, particularly in the non-transplant setting, as they are present in all tissues of the host and therefore not disease-specific. Analyses were performed using default settings, with the following exceptions: no sub-sampling of reads, maximum read number 10 × 10^6^ (to include all reads), minimal depth for variant call = 1, error-correction: on. Mutations that were previously detected by routine diagnostics but that did not pass automatic filtering were manually revisited in the data set. Mutations that passed automatic filtering but had not been reported by the reference laboratory were analyzed in concordance to the joint ClinGen, CGC and VICC guidelines^22^. Recurrent AML-associated mutations were identified by cross-referencing clinical databases such as COSMIC^23^ and ClinVar^24^. Computational evidence was gathered using the following algorithms: FATHMM^25^, PolyPhen2^26^, PROVEAN^27^ und SIFT^28^. Only mutations rated as likely pathogenic or pathogenic were included in further analyses.

### CR assessment and chimerism analysis

CR was defined as detection of < 5% blasts in bone marrow biopsies as determined by routine cytology and not further distinguished from incomplete CR without complete regeneration of peripheral blood counts. A patient was considered to be in CR at any time point after documentation of CR where there was no clinical suspicion of relapse. In patients that had received aHSCT, donor chimerism was determined by genotyping of 12 short-tandem-repeat loci and the amelogenin locus using the MENTYPE Chimera Kit (Biotype, Dresden, Germany) as described before^29^. In patients whose main blast population was CD34-positive at initial diagnosis, chimerism analysis was performed on magnetically enriched CD34 + cells and in other patients in total white blood cells as described previously^5^. For the purpose of this study, we did not discriminate between CD34- and total chimerism and considered a donor chimerism < 90% as indicative for relapse instead of separate thresholds (< 80% for CD34- and < 94% for total chimerism^5^.

### Statistical analysis

Statistical analysis was performed using GraphPad Prism versions 7 and 8 (GraphPad Software, San Diego, CA, USA). Details on the particular tests used are specified in the figure legends. *p* < 0.05 was considered as statistically significant.

## Results

### Patient characteristics and AML mutational spectrum

This study included 29 patients with AML who received treatment as appropriate to their disease and were followed-up at our center. Baseline-characteristics of this patient group are summarized in Table 1 and Supplementary Table S4. The median number of AML-related mutations present in each patient upon initial diagnosis was 2 (range 1–7) and a total of 75 mutations affecting 16 different genes were detected in these 29 patients. The most frequently affected genes in this patient group were NPM1 (mutated in 38% of patients), DNMT3A (28%) and IDH2 (24%; Supplementary Table S5).

**Table 1:** Patient characteristics.

Total number of patients, *n* (%)	29 (100)
Male	7 (24.1)
Female	22 (75.9)
< 65 years at initial diagnosis	24 (82.7)
> 65 years at initial diagnosis	5 (17.3)
Age at initial diagnosis in years, median (range)	57 (17–82)
*AML subtype according to WHO classification (2022)*,* n (%)*	
AML post cytotoxic therapy*	2 (6.9)
AML with defining genetic abnormalities	24 (82.3)
AML with *NPM1* mutation	11 (37.9)
AML with *BCR::ABL1* fusion	1 (3.4)
AML, myelodysplasia-related	12 (41.4)
AML, defined by differentiation	3 (10.3)
AML with minimal differentiation	1 (3.4)
Acute myelomonocytic leukemia	1 (3.4)
Acute monocytic leukemia	1 (3.4)
*Risk stratification according to ELN (2022)*,* n (%)*	
Favorable	8 (27.6)
Intermediate	5 (17.2)
Adverse	16 (55.2)
Pts. receiving an allogeneic stem cell transplant during course of therapy, n (%)	25 (86.2)

*including one patient with *KMT2A*-rearrangement.

### CfDNA in AML patients

**Fig. 1:**
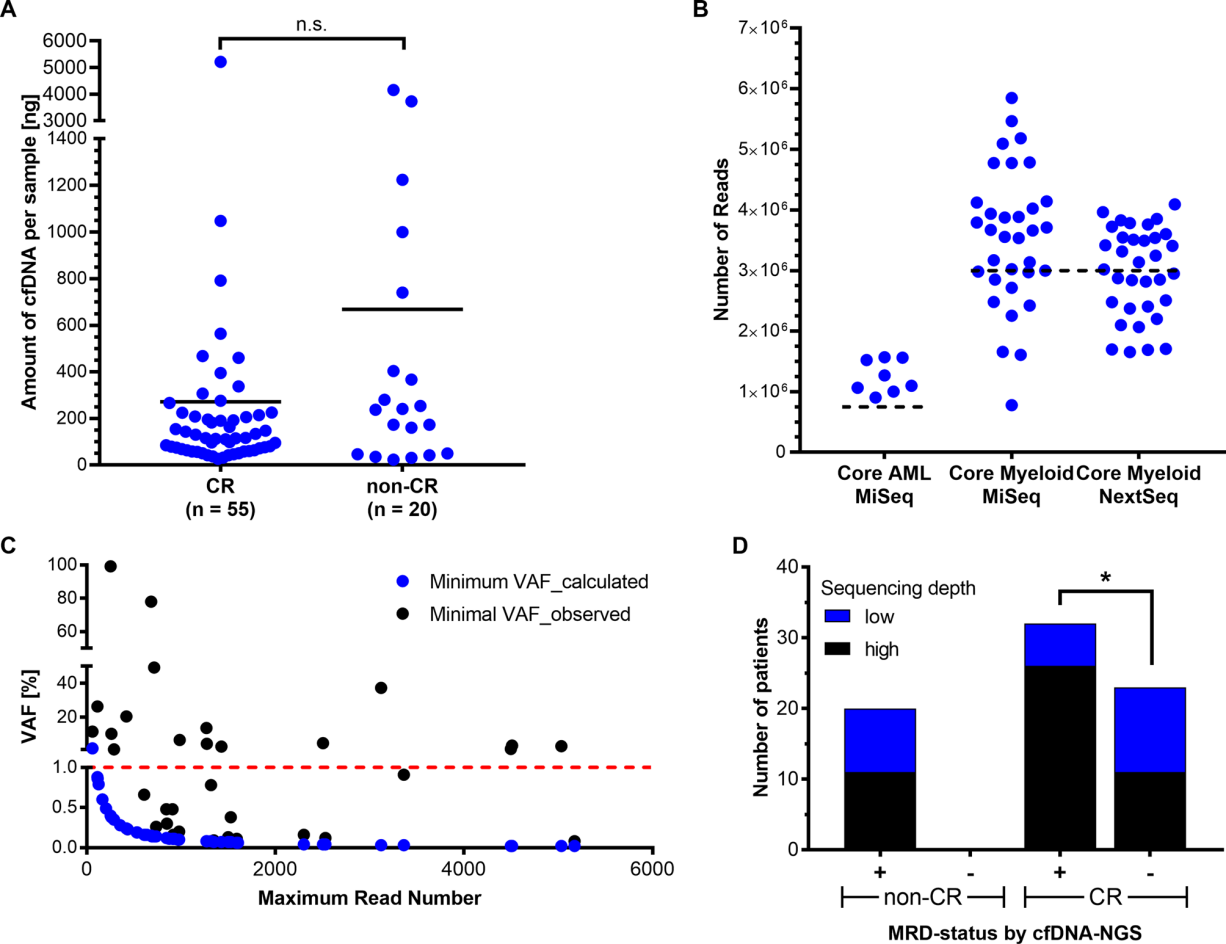
NGS of cfDNA samples in AML patients. (**A**) Amount of cfDNA isolated from peripheral blood samples of AML patients. cfDNA-levels in CR samples (*n* = 55) were not significantly different from non-CR samples (*n* = 20) (*p* = 0,1705; Mann-Whitney-U-Test). Horizontal lines indicate the median in each group. N.s. not significant. (**B**) Sequencing depth obtained for 75 cfDNA samples of AML patients grouped by sequencing panels and instruments. Sample numbers are *n* = 8 for Core AML/MiSeq, *n* = 33 for Core Myeloid/MiSeq and *n* = 34 for Core Myeloid/NextSeq. Horizontal dotted lines indicate the recommended depth for the respective panel (Core AML: 750.000; Core Myeloid: 3.000.000). (**C**) Theoretical sensitivity (i.e. lowest detectable VAF in our experiments; calculated as 1/max. read number at the mutational site) and lowest observed VAF for mutations detected in AML cfDNA samples. The red line indicates the MRD threshold, corresponding to a VAF of 1%. (**D**) MRD status by cfDNA-NGS and sequencing depth. Low and high sequencing depth were defined as NGS read number below and above, respectively, the minimum read number recommended for panel analysis. Patients were grouped based on CR-status by routine diagnostics. Contingency in non-CR or CR patients was assessed by Fisher’s exact test. (*p* = 0.0184). See also Supplementary Table S7.

The minimum input recommended by the manufacturer for the NGS-panel-assays used in this study is 10 ng of DNA. cfDNA concentrations obtained from one 10 ml blood collection tube per patient and timepoint ranged from 0.592 to 130.4 ng/µl, corresponding to 24 ng to 5.2 µg per patient sample. The median amount of cfDNA in samples taken during CR was 116 ng compared to 239.2 ng in non-CR samples, although this difference was not statistically significant (*p* = 0.1705; Fig. 1A). All samples passed pre-sequencing quality controls. Thus, cfDNA could be isolated in sufficient quantity and quality for molecular analyses by NGS in AML patients irrespective of disease state at the time of sampling.

### Sequencing depth and sensitivity of mutation detection

Next we assessed the ability of the off-the-shelf panel-sequencing assays to detect mutations in CR and non-CR-samples from AML patients based on whether the recommended sequencing depth had actually been obtained or not. In all sequencing runs, flow cells were loaded so that the number of sequencing reads required for optimal sensitivity indicated in the manufacturer’s protocol could be reached. 48 of 75 samples (64%) yielded at least the required number of reads, including 30 of 41 samples (73%) sequenced on the MiSeq and 18 of 34 samples (53%) of samples sequenced on the NextSeq (Fig. 1B). This observation indicates that sequencing experiments on larger instruments incorporating a high number of samples are more sensitive to imbalances of sample-concentrations/-amounts within the sequencing library-pool. Analysis of sequencing depth at mutational hotspots revealed that 88% of these sites were covered at least 100x in samples where these mutations were known to be present (Supplementary Table S6). Thus, assay sensitivity was 10^−2^ for the vast majority of mutations, which was below the lowest VAF observed for each mutation in our sample series (Fig. 1C). These findings confirm that the method applied in this study is in principle suitable for assessment of MRD in a routine clinical setting.

### AML-associated mutations CfDNA in CR and non-CR samples

Having analyzed the sensitivity of cfDNA-based mutation detection in AML according to sequencing depth, we further investigated the presence of AML-specific mutations depending on disease state. A sample was considered positive for residual disease if at least one mutation identified at initial diagnosis in the respective patient was detectable in cfDNA. 32 of 55 samples (58%) taken during CR were mutation-positive, including 26/37 samples (70%) that had been sequenced with high depth and 6/18 samples (33%) that had been sequenced with an insufficient number of total reads (*p* = 0.0184, Fisher`s exact test). On the other hand, all 20 non-CR samples (100%) revealed evidence of disease, although only 11 of these samples (55%) had yielded the recommended number of reads (Fig. 1D, Supplementary Table S7, Supplementary Figure S1). In total, 35 of the 51 mutations (43%) that had been reported by the reference laboratory at initial diagnosis were detectable in CR and non-CR samples, with 15 mutations being present in CR, 11 in non-CR and 9 in both CR and non-CR samples (Supplementary Tables S7, S8). Together, these findings underline that optimal sequencing depth is essential to obtain valid results from cfDNA studies in the context of MRD-analyses, i.e. in AML patients in hematologic CR.

### VAF of detectable and non-detectable mutations in CfDNA in CR and non-CR samples

**Fig. 2:**
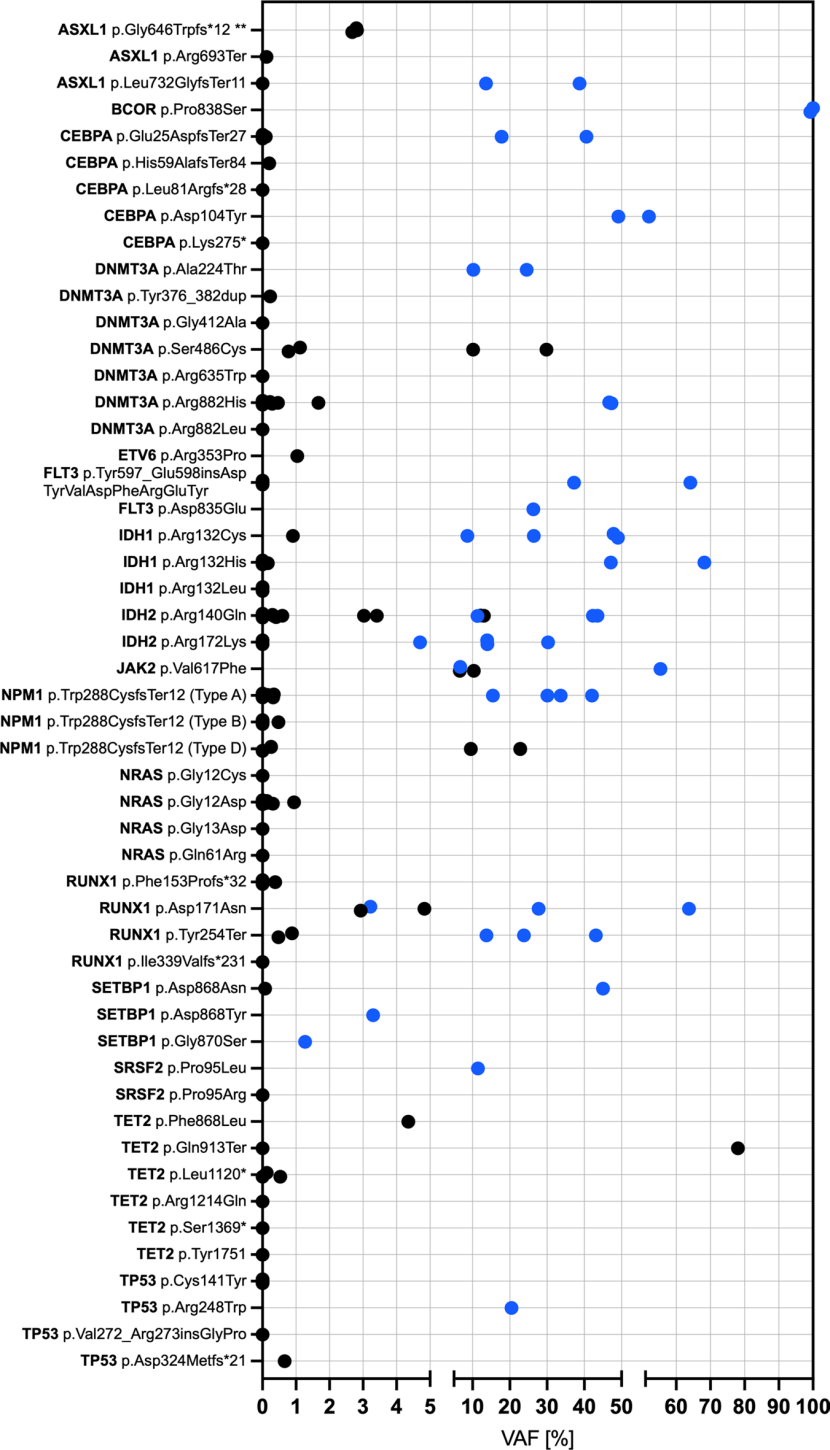
VAF of AML-associated mutations in cfDNA of AML patients. Samples obtained at time points when patients were in complete hematological remission are indicated in black, non-CR samples are indicated in blue.

To examine the burden of residual disease as indicated by the presence of AML-specific mutations in a quantitative manner, we compared variant allele frequencies (VAF) in CR and non-CR samples and investigated in detail which mutations had been missed by cfDNA analysis. As expected, VAF was lower in CR-samples (0.08–78.04%) than in non-CR samples (1.27–100%), with 32 of 53 mutations (60.4%) detectable in CR displaying a VAF < 1% and all mutations present at non-CR showing a VAF > 1% (Fig. 2).

30 samples (13 CR, 17 non-CR) contained all mutations identified at initial diagnosis in the respective patient, while only a fraction of previously reported variants could be recovered in 22 cfDNA-samples (19 CR, 3 non-CR). In 10 of these 22 samples (45.5%), non-detectable mutations had lower VAF at initial diagnosis than still detectable mutations, indicating that these variants were subclonal and potentially eradicated by therapy. The three non-CR samples which did not contain the full mutation spectrum described at initial diagnosis were from the same patient, and the non-detectable mutation was a *FLT3*-ITD. Of note, one of these samples was taken at initial diagnosis 8 days before a BM sample, in which the *FLT3*-ITD was detected at a high ratio (0.602). These findings suggest, that NGS of cfDNA allows for more reliable quantification of disease burden when short sequence variants are analyzed, while the method appears to be prone to overlook *FLT3*-ITDs due to technical and/or bioinformatic issues.

### Correlation of VAF in CfDNA and cellular DNA

**Fig. 3:**
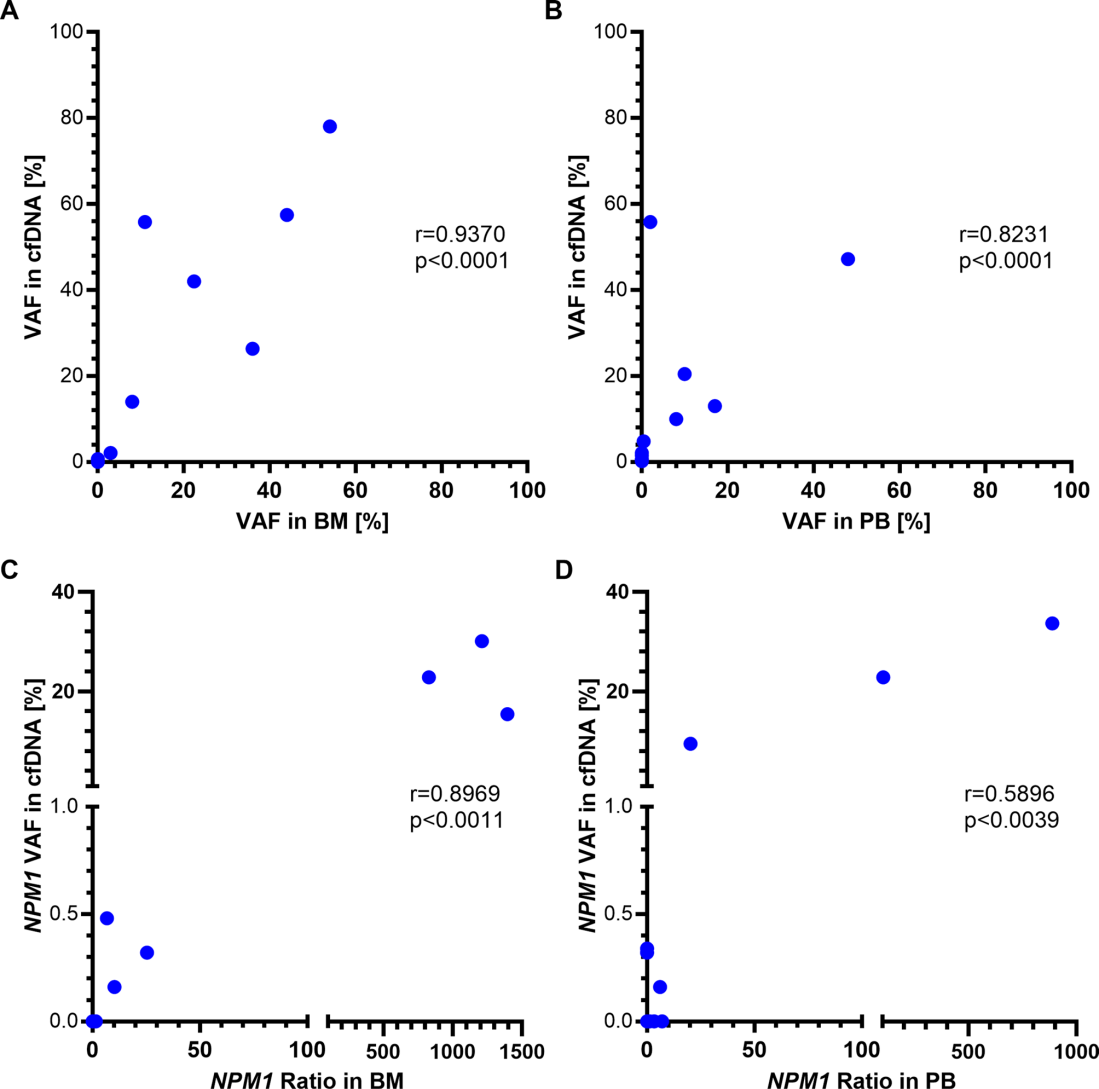
Mutational load in cfDNA and cellular DNA from the bone marrow or peripheral blood of AML patients. (**A**, **B**) Correlation of VAF as determined by NGS of cfDNA and VAF from NGS-analysis of BM (**A**) or PB (**B**) samples reported by the reference laboratory. (**A**) *n* = 12, Spearman correlation *r* = 0.9370, *p* < 0.0001. (**B**) *n* = 17, Spearman correlation *r* = 0.8231, *p* < 0.0001. (**C**, **D**). Correlation of VAF of NPM1-mutations as determined by NGS of cfDNA and NPM1-mutation ratio determined by qPCR of BM (**C**) or PB (**D**) by the reference laboratory. (**C**) *n* = 10, Spearman correlation *r* = 0.8969, *p* = 0.0011. (**D**) *n* = 22, Spearman correlation *r* = 0.5896, *p* = 0.0039. Note that in *n* = 3 (BM) and *n* = 5 (PB) patients, respectively, more than one sample pair per patient was included in the analysis).

Given that quantification of AML disease-burden by NGS of cfDNA may not be equally suitable for all potential clonal or subclonal disease markers, we next compared our experimental method to routine disease monitoring. First, we analyzed VAFs of mutations in cfDNA and in reference samples from the bone marrow or peripheral blood that had been taken at similar time points, i.e. within 30 days before or after cfDNA-sampling, and had been examined by NGS panel-analysis by the reference laboratory. 12 BM and 17 PB samples were available for this analysis, and only mutations present in both cfDNA and reference samples were considered. In cases with more than one mutation, mean VAFs were analyzed. We observed significant positive correlations between VAFs in cfDNA and BM or, respectively PB (BM: *r* = 0.9370, *p* < 0.0001, Fig. 3A, PB: *r* = 0.8231, *p* < 0.0001, Fig. 3B), suggesting that disease burden determined by NGS in cfDNA is indicative for residual AML in both compartments.

### Detection of NPM1-mutations by cfDNA-NGS

In addition, we focused on NPM1-mutations, for which the preferred method of MRD-quantification in current clinical routine is qPCR rather than NGS. NPM1-mutations are among the most common mutations observed in AML patients and had been reported at initial diagnosis in 11 of 29 patients in our cohort. 10 matched pairs of cfDNA and BM-samples (taken within 21 days) and 22 pairs of cfDNA and PB from 10 patients were available for comparison of disease burden. NPM1-mutations were detected in 8/10 bone marrow-samples by qPCR and in 6/10 cfDNA-samples (Supplementary Table S9). Two pairs were negative by both methods and two pairs yielded discordant results. In PB, NPM1-mutations were detected in 10/22 samples by qPCR, and 5 of these 10 samples were also positive for NPM1-mutations in cfDNA, while 12 sample pairs were negative and 6 were positive by only one method. The VAF in cfDNA correlated well with the qPCR ratio in BM (Spearman correlation; *r* = 0.8969, *p* = 0.0011; Fig. 3C). In PB, NPM1-mutation VAF correlated moderately with the qPCR ratio (Spearman correlation; *r* = 0.5896, *p* = 0.0039, Fig. 3D).

In sample pairs in which the NPM1 mutation was detected by qPCR, but not by NGS, the NPM1 ratio was low (0.146–1.523 in BM, 0.116–7 in PB). Beyond that, two cfDNA samples in which the NPM1 mutation could not be detected (corresponding to BM reference samples) had been sequenced with insufficient coverage. When we analyzed the presence of NPM1-mutations in cfDNA according to disease state, we found that only 9 of 29 CR-samples (31%), but all 3 non-CR-samples were positive (Supplementary Table S9). In summary, these observations suggest, that for detection of NPM1-mutations in the setting of MRD, standard qRT-PCR from a cellular source of DNA is more sensitive than cfDNA-analysis by NGS.

### Monitoring of AML after therapy by NGS of CfDNA

**Fig. 4:**
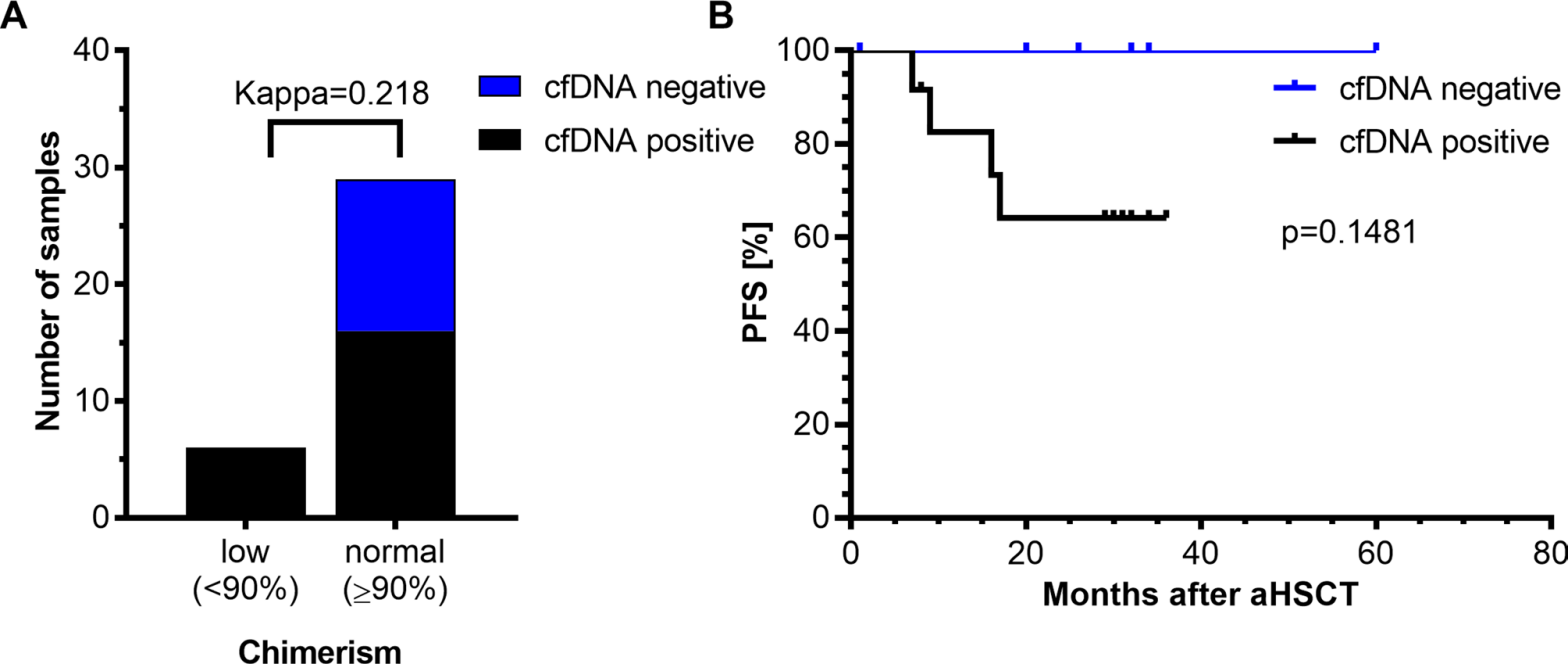
Detection of residual/relapsed AML by cfDNA-NGS and donor chimerism analysis in patients after aHSCT. (**A**) Detection of AML in cfDNA compared to chimerism (*n* = 35). Normal/low chimerism was defined as chimerism ≥ 90% and < 90% respectively. Interrater agreement of the two methods to reveal evidence of AML was assessed by Cohen’s Kappa (95% CI = 0.042–0.394. (**B**) Progression-free survival of patients with normal donor chimerism. PFS-data was available for 18 patients with normal donor chimerism and patients were grouped based on cfDNA-mutation status. Patients for whom at least one AML-specific mutation described at initial diagnosis was identified in cfDNA were considered MRD positive (*p* = 0.1481, Log-Rank-Test).

As the analyses conducted so far clearly suggested that examination of cfDNA for AML-related mutations was in principle suitable to detect MRD, we investigated whether disease state indicated by cfDNA was predictive for relapse after aHSCT. aHSCT was applied as post-remission therapy in 25 of the 29 patients, while four patients were treated by chemotherapy only. Of the four patients, three relapsed between 302 and 665 days after diagnosis, while one patient died 54 days after diagnosis before starting consolidation therapy (Table 1, Table S2). Routine follow-up after aHSCT included analysis of donor chimerism in the peripheral blood for early detection of imminent relapse. 35 time-matched pairs (taken a maximum of 21 days apart from each other) of PB and cfDNA samples obtained from 22 patients at up to three timepoints after aHSCT were available for comparative analysis of disease state by both methods. AML-related mutations were detected in all 6 cfDNA-samples that corresponded to blood samples that displayed decreased donor chimerism (< 90%). Moreover, 16 of 29 cfDNA samples that corresponded to blood samples with normal donor chimerism exhibited mutations (Fig. 4A, Supplementary Table S10). The median donor chimerism at the time of MRD positivity in CR samples was 98% (range: 93–99%) (Supplementary Figure S2). Thus, there was only fair agreement of the two methods, chimerism and cfDNA analysis, to detect residual/relapsed disease (Kappa = 0,218, 95%-CI 0,042 − 0,394). This observation raises the possibility that mutational status in cfDNA defines subgroups of patients with normal donor chimerism after aHSCT with diverse clinical courses. When patients with normal donor chimerism were grouped by presence or absence of AML-related mutations in cfDNA, Kaplan-Meier analysis indicated a trend towards shorter progression-free survival in the mutation-positive subgroup, although this trend did not reach statistical significance (Fig. 4B). In patients who were treated by chemotherapy only, 8 cfDNA samples (4 CR, 4 non-CR) were available, all of which displayed at least a subset of AML-related mutations reported previously by the reference laboratory. In fact, 7 of 8 samples included the complete spectrum of mutations. These findings confirm, that NGS of cfDNA is suitable for monitoring AML disease burden under/after therapy and raise the possibility that MRD-positivity is prognostically relevant, at least in the setting of normal donor chimerism after aHSCT.

### Clonal evolution in CfDNA from AML patients

**Fig. 5:**
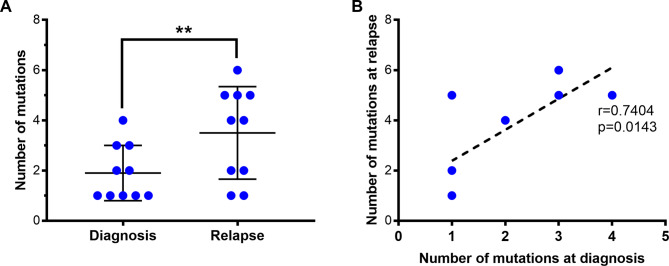
Clonal evolution in AML patients as indicated by NGS of cfDNA. *N* = 10 patients, for whom cfDNA samples were available close to relapse. (**A**) Number of mutations at initial diagnosis as reported by the reference laboratory from BM analyses compared to number of mutations detected in cfDNA at relapse. Black lines indicate means with standard deviation (*p* = 0.0031; paired t-test). (**B**) Pearson correlation of mutational load at initial diagnosis and relapse (*r* = 0.7404; *p* = 0.0143).

Given our preliminary evidence that MRD as determined by NGS of cfDNA may be predictive for relapse, we explored whether the method was also capable of detecting clonal evolution, which is frequently observed in AML at relapse. Among the 29 patients included in this study, 17 experienced a relapse of their AML (Table 1). To determine whether cfDNA-NGS allows for the detection of newly emerged subclones that may have contributed to relapse, we analyzed 10 patients for which cfDNA samples had been prepared close to relapse (maximum of 33 days before/after). In 8/10 cfDNA-samples novel mutations, i.e. mutations not detected upon initial diagnosis, were identified and median mutation burden was three mutations (range 1–6) compared to 1,5 mutations (range 1–4) per patient upon initial diagnosis. More precisely, the number of mutations detected at relapse was significantly higher than the number of mutations at initial diagnosis (*p* = 0.0031, Fig. 5A) and there was a significant correlation between mutational loads at initial diagnosis and at relapse (Pearson correlation *r* = 0.7404, *p* = 0.0143, Fig. 5B). In the seven patients that displayed new mutations after relapse, the most frequently affected genes were DNMT3A (2/8; 25%), CBL (2/8; 25%) and TET2 (2/8; 25%). Only in one patient, an *FLT3*-ITD detected at initial diagnosis was no longer detectable upon relapse (Supplementary Table S11). Of note, since we had no access to the raw data of molecular analyses conducted in the reference laboratory and NGS analyses were not performed routinely at relapse at the time this pilot study was conducted, we cannot exclude that all mutations detected in cfDNA at relapse were actually present in BM/PB at relapse and/or at initial diagnosis with a low VAF below the threshold for reporting. Most of the novel mutations had a VAF > 5%, but in one patient, an NRAS mutation that had been confirmed by the reference laboratory was present at a very low VAF of 0.08%, underlining our hypothesis that these were valid mutations rather than artifacts. Therefore, our results clearly indicate that monitoring AML-related mutations in cfDNA can detect biological changes throughout the course of the disease.

## Discussion

Here, we adapted a commercial NGS-assay for mutational screening in myeloid malignancies for MRD-monitoring in AML based on cfDNA. Our results have important clinical implications as they suggest our approach may be useful to better stratify relapse risk in patients with normal donor chimerism after aHSCT, even though further studies are necessary to overcome technical difficulties and limitations of our work.

First, there was no fixed schedule for sample collection and parallel monitoring of mutations in BM and/or PB was not performed routinely at the time our pilot study was conducted. Thus, our sample series is quite heterogenous, which impairs statistical testing. Moreover, recommended sequencing depth was not reached in all samples, very likely compromising the sensitivity of the assay for these cases. The recommended sequencing depth, as we clearly show, is appropriate to detect mutations at a VAF in the MRD range, i.e. <5% and even < 0,1%. Sensitivity was variable for different mutations, also consistent with recent publications that reported sensitivities of 10^−2^ to 10^−6^ for NGS-based MRD detection^30^. Additionally, our results corroborate a recent systematic review that provided evidence for improved disease-free and overall survival in AML patients who achieve MRD negativity, regardless of the sensitivity threshold and across different detection methods such as MFC and qPCR^31^. Of note, limited amount of input was not an issue in our study, as we were able to isolate sufficient cfDNA for NGS from a single blood collection tube in all patients. Consistent with previous studies, levels of cfDNA were higher in non-CR samples than in CR-samples^32,33^.

Beyond that, our results underline two more general technical challenges of NGS compared to other molecular methods. In particular, detection of *FLT3*-ITDs is not reliable by targeted sequencing of cfDNA since this mutation was missed in one patient even in the cfDNA sample that had been collected at initial diagnosis. In fact, *FLT3*-ITDs as determined by NGS have been used to quantify MRD in AML and have been attributed prognostic significance^34–36^. However, one previous study excluded some patients, in whom the *FLT3*-ITD could be detected only by fragment length analysis, but not by NGS at initial diagnosis^35^. These observations point out, that current NGS and bioinformatics methods cannot fully replace other laboratory tests for mutation screening in AML, as *FLT3*-ITDs represent therapeutic targets already in the first line of treatment that must not be overlooked^37,38^.

In addition, NGS of cfDNA in our work did not detect mutations in NPM1, the best established MRD marker in AML^39^, in two samples which had been positive by qPCR-based reference diagnostics. The sensitivity of our assay allowed for detection of mutations down to a VAF of approximately 10^−2^, in line with previous reports^40^ and it may be assumed that this constraint caused false negative results in cfDNA MRD assays. However, discordant results between NGS and standard qPCR for NPM1-MRD have been observed by others despite the use of cellular DNA as an input and higher mutation-specific coverage enabling a sensitivity of 10^−4^^41^. Only standard NPM1-MRD-monitoring, but not NGS-MRD was associated with shortened overall survival in multivariate analysis in that publication. This notion also justifies our straight-forward technical approach that did not involve assay optimization such as maximizing the amount of input or the number of sequencing reads, which might have improved the detection of NPM1-mutations and also of *FLT3*-ITDs^42–44^. Irrespective of technical issues, failure to detect mutations by cfDNA sequencing may also be explained by clonal heterogeneity of a given patient’s AML, as previous studies in both adult and pediatric AML have shown that the mutational spectrum detectable by NGS in BM and cfDNA is not fully congruent^40,45^. Thus, analysis of cfDNA is also capable of detection of isolated extramedullary relapse, further supporting the biological rationale of incorporating cfDNA into clinical routine diagnostics^46^.

Despite these limitations, our study has highly relevant clinical implications. Specifically, our data strongly suggest that NGS of cfDNA can distinguish subgroups of patients after aHSCT with normal donor chimerism, but different prognosis depending on the presence or absence of AML-related mutations. We attribute the fact that shorter PFS in patients with positive cfDNA was not statistically significant to the small number of samples available for this analysis. Nonetheless, our observations corroborate previous work that reported increased relapse rates in AML/MDS patients with persistent disease-associated mutations after aHSCT compared to patients who were cfDNA negative^17,32^. Moreover, we provide preliminary evidence that NGS of cfDNA is suitable to uncover clonal evolution of AML. Importantly, current guidelines for molecular MRD analyses in AML emphasize that variants not found at diagnosis should be reported only if they can be distinguished clearly from background^19^. Following this recommendation, we found a higher number of mutations in cfDNA at relapse than at initial diagnosis. Most of the mutations that were present at both initial diagnosis and relapse affected genes that are known indicators of clonal hematopoiesis of indeterminate potential (CHIP), such as *ASXL1*, *DNMT3A*, and *TET2*^47^. Previous studies of MRD in AML patients in CR have shown, that CHIP-associated mutations persist in > 46–78% of cases^48,49^. The prognostic impact of persisting CHIP mutations may be different in various clinical contexts, specifically in first remission and in the post-transplant setting, since failure to clear clonal hematopoiesis after aHSCT has recently been found to be associated with inferior outcomes^48–51^. Although these previous studies investigated cellular DNA, these observations are very likely valid for cfDNA, which is mainly derived from the hematopoietic system. Finally, from a practical laboratory perspective, monitoring MRD in cfDNA in addition to chimerism means additional costs and efforts and the approach described here needs to be validated against state-of-the art technologies for chimerism analysis such as NGS-based assays, which have recently been described to enable both greater sensitivity and accuracy than traditional methods^52^. Compared to other molecular methods of mutation detection, targeted NGS of cfDNA is approximately equally expensive and time-consuming than NGS of bone marrow or peripheral blood DNA with a turnaround time of 3–5 days, but apparently more costly and laborious than a single-target qPCR assay, which can be performed overnight.

## Conclusions

In summary, the study presented here substantially adds to previous work on the use of cfDNA to monitor AML. Our results confirm that a one-fits-all NGS approach is not the preferred assay for all AML-related mutations and that cellular and cell-free DNA may not be interchangeable, but rather complimentary to capture the full heterogeneity of AML. As longitudinal sequencing of cfDNA only involves a minimally invasive sampling procedure, the approach offers the perspective to shorten follow up intervals in selected high-risk patients, particularly after aHSCT, provided that the clinical implications suggested here are confirmed by further studies.

## Supplementary Information

Below is the link to the electronic supplementary material.

Supplementary Material 1

## Data Availability

Raw data from sequencing experiments are available from the corresponding author upon reasonable request.

## Abbreviations

AML: Acute myeloid leukemia
CHIP: Clonal hematopoiesis of indeterminate potential
CR: Complete remission
MRD: Measurable/minimal residual disease
NGS: Next generation sequencing
q(RT-)PCR: Quantitative (reverse-transcription) polymerase chain reaction
