# Supplementary material for: Detection of minimal residual disease in circulating cell-free DNA in acute myeloid leukemia
Source: Sci Rep. 2025 Sep 23;15:32679. doi: 10.1038/s41598-025-20589-3 (PMC12457635; doi:10.1038/s41598-025-20589-3)
Supplement: Supplementary file 1 — Supplementary Material 1 [file 41598_2025_20589_MOESM1_ESM.pdf]

Detection of minimal residual disease in circulating cell-free DNA in Acute Myeloid Leukemia

Supplementary Materials

Charlotte Sommer[1], Hildegard I. D. Mack[1], Madeleine C. Killer[1], Petra Ross[1], Andrea Nist[2], Thorsten Stiewe[2,3], Andreas Neubauer[1], Cornelia Brendel[1], and Elisabeth K. M. Mack[1, 4]*

1 Department of Hematology, Oncology and Immunology, Philipps-University Marburg, and University Hospital Gießen and Marburg, Campus Marburg, Marburg, Germany.

2 Genomics Core Facility, Institute of Molecular Oncology, Universities of Gießen and Marburg Lung Center, Member of the German Center for Lung Research (DZL), Philipps-University Marburg, Marburg, Germany.

3 Institute of Lung Health, University Gießen, Germany.

4 Present address: Department of Hematology, Medical Oncology and Palliative Medicine, St. Marien-Krankenhaus Siegen, Siegen, Germany

Supplementary Table S1: Overview of reference samples in relation to cfDNA sample collection.

Patient	Sample ID (cfDNA)	Timepoints of cfDNA sample collection after initial diagnosis / alloSCT (days)	Timepoints of bone marrow biopsies after ID / alloSCT (days)*	Time after last documented bone marrow biopsy indicating CR/non-CR before cfDNA sampling (days)***	Timepoints of chimerism analysis after ID / alloSCT (days)**	Time from/to chimerism analysis closest to cfDNA sampling (days)***
1	131917	178 / 112	150 / 84	28	178 / 112	0
2	122517	119 / 63	87 / 31	32	129 / 73	10
	15718	203 / 147	-	116	177 / 121	26
3	131117	196 / 108	64 / NA (before alloSCT)	132	182 / 94	14
	136417	210 / 122	-	146	-	28
	9018	245 / 157	-	181	252 / 164	7
	56018	371 / 283	-	307	336 / 248	35
	120618	546 / 458	-	482	546 / 458	0
4	11818	5 / NA (before alloSCT)	0 / NA (before alloSCT)	5	NA (before alloSCT)	NA (before alloSCT)
	35718	69 / NA (before alloSCT)	60 / NA (before alloSCT)	9	NA (before alloSCT)	NA (before alloSCT)
	75418	175 / 84	175 / 84	0	158 / 67	17
	117218	291 / 200	-	116	284 / 193	7
	19719	389 / 298	375 / 284	14	350 / 259	39
5	14917	50 / NA (before alloSCT)	0 / NA (before alloSCT)	50	NA (before alloSCT)	NA (before alloSCT)
	111117	303 / 198	246 / 141	57	303 / 198	0
	17518	414 / 309	331 / 226	83	398 / 293	16
	56718	524 / 419	500 / 395	24	566 / NA (after relapse, before 2nd alloSCT)	NA (after relapse, before 2nd alloSCT)
	117318	692 / 145 (after 2nd alloSCT)	637 / 90	54	625 / 78 (after 2nd alloSCT)	67
6	137817	281 / 206	36 / NA (before alloSCT)	245	267 / 192	14
7	130917	105 / 55	105 / 55	0	81 / 31	24

Patient	Sample ID (cfDNA)	Timepoints of cfDNA sample collection after initial diagnosis / alloSCT (days)	Timepoints of bone marrow biopsies after ID / alloSCT (days)*	Time after last documented bone marrow biopsy indicating CR/non-CR before cfDNA sampling (days)***	Timepoints of chimerism analysis after ID / alloSCT (days)**	Time from/to chimerism analysis closest to cfDNA sampling (days)***
8	74317	73 / NA (before alloSCT)	64 / NA (before alloSCT)	9	NA (before alloSCT)	NA (before alloSCT)
	129117	209 / 131	-	145	195 / 117	14
	18818	286 / 208	-	222	223 / 145	63
	81818	461 / 383	-	397	-	238
9	127917	437 / 339	71 / NA (before alloSCT)	366	437 / 339	0
10	47618	154 / 62	133 / 41	21	144 / 52	10
11	126218	165 / 116	132 / 83	33	175 / 126	10
	3319	203 / 154	200 / 151	3	207 /158	4
	19619	242 / 193	-	42	263 / 214	21
	52519	347 / 298	270 / 221	77	355 / 306	8
12	19817	51 / NA (before alloSCT)	45 / NA (before alloSCT)	6	NA (before alloSCT)	NA (before alloSCT)
	27017	84 / NA (before alloSCT)	-	39	NA (before alloSCT)	NA (before alloSCT)
	44817	133 / 21	91 / NA (before alloSCT)	42	133 / 21	0
	108717	296 / 184	-	205	296 / 184	0
	4618	387 / 275	-	296	335 / 223	52
	108018	667 / 555	-	576	-	332
13	25518	255 / NA (before alloSCT)	255 / NA (before alloSCT)	0	NA (before alloSCT)	NA (before alloSCT)
	57218	340 / NA (before alloSCT)	340 / NA (before alloSCT)	0	NA (before alloSCT)	NA (before alloSCT)
	81718	409 / 31	-	69	412 / 34	3
14	22618	63 / NA (before alloSCT)	63 / NA (before alloSCT)	0	NA (before alloSCT)	NA (before alloSCT)
	30118	85 / NA (before alloSCT)	-	18	NA (before alloSCT)	NA (before alloSCT)
	21819	427 / 336	-	357	392 / 301	35

Patient	Sample ID (cfDNA)	Timepoints of cfDNA sample collection after initial diagnosis / alloSCT (days)	Timepoints of bone marrow biopsies after ID / alloSCT (days)*	Time after last documented bone marrow biopsy indicating CR/non-CR before cfDNA sampling (days)***	Timepoints of chimerism analysis after ID / alloSCT (days)**	Time from/to chimerism analysis closest to cfDNA sampling (days)***
15	35018	1032 / 155 (after 2nd alloSCT)	1029 / 152 (after 2nd alloSCT)	3	1029 / 152 (after 2nd alloSCT)	3
16	137217	394 / NA (no alloSCT)	375 / NA (no alloSCT)	19	NA (no alloSCT)	NA (no alloSCT)
17	88018	574 / NA (no alloSCT)	519 / NA (no alloSCT)	55	NA (no alloSCT)	NA (no alloSCT)
	111018	644 / NA (no alloSCT)	-	123	NA (no alloSCT)	NA (no alloSCT)
	22519	763 / NA (no alloSCT)	738 / NA (no alloSCT)	25	NA (no alloSCT)	NA (no alloSCT)
	29119	781 / NA (no alloSCT)	-	43	NA (no alloSCT)	NA (no alloSCT)
18	114318	229 / NA (no alloSCT)	88 / NA (no alloSCT)	141	NA (no alloSCT)	NA (no alloSCT)
	22419	340 / NA (no alloSCT)	302 / NA (no alloSCT)	38	NA (no alloSCT)	NA (no alloSCT)
19	126017	735 / 46	675 / (before alloSCT)	60	731 / 42	4
	81318	990 / 301	794 / 105	196	990 / 301	0
	24619	1207 / 515	-	410	1207 / 515	0
20	8218	1374 / 1295	1374 / 1295	0	1374 / 1295	0
21	124417	778 / 64 (after 2nd alloSCT)	774 / 60 (after 2nd alloSCT)	4	760 / 46 (after 2nd alloSCT)	18
22	11118	7 / NA (before alloSCT)	0 / NA (before alloSCT)	7	NA (before alloSCT)	NA (before alloSCT)
	22718	31 / NA (before alloSCT)	-	31	NA (before alloSCT)	NA (before alloSCT)
	21719	395 / 280	-	220	374 / 259	21
23	25118	3573 / 262	3263 / NA (before alloSCT)	310	3573 / 262	0
24	58718	249 / 117	NA (BM biopsy was not done; blood showed elevated blast count)	NA (BM biopsy was not done; blood showed elevated blast count)	238 / 106	11
25	112918	228 / 103	208 / 83	20	228 / 103	0

Patient	Sample ID (cfDNA)	Timepoints of cfDNA sample collection after initial diagnosis / alloSCT (days)	Timepoints of bone marrow biopsies after ID / alloSCT (days)*	Time after last documented bone marrow biopsy indicating CR/non-CR before cfDNA sampling (days)***	Timepoints of chimerism analysis after ID / alloSCT (days)**	Time from/to chimerism analysis closest to cfDNA sampling (days)***
	126118	256 / 131	250 / 125	6	270 / 145	14
	119	286 / 161	-	36	-	16
	21619	336 / 211	-	86	305 / 180	31
26	33017	50 / NA (before alloSCT)	48 / NA (before alloSCT)	2	NA (before alloSCT)	NA (before alloSCT)
	60518	489 / 335	114 / NA (before alloSCT)	375	489 / 335	0
27	13617	358 / NA (before alloSCT)	333 / NA (before alloSCT)	25	NA (before alloSCT)	NA (before alloSCT)
	23117	386 / NA (before alloSCT)	-	53	NA (before alloSCT)	NA (before alloSCT)
28	22917	1 / NA (before alloSCT)	1 / NA (before alloSCT)	0	NA (before alloSCT)	NA (before alloSCT)
	45517	63 / NA (before alloSCT)	16 / NA (before alloSCT)	47	NA (before alloSCT)	NA (before alloSCT)
	75717	139 / 21	92 / NA (before alloSCT)	47	138 / 20	1
	102717	212 / 94	-	120	212 / 94	0
	1118	309 / 191	-	217	323 / 205	14
	114418	615 / 497	614 / 496	1	463 / 345	152
29	26117	21 / NA (no alloSCT)	20 / NA (no alloSCT)	1	NA (no alloSCT)	NA (no alloSCT)

* Only the bone marrow samples relevant for determining the remission status at the time of cfDNA sampling are listed. A "-" indicates that no more recent bone marrow sample was taken prior to the corresponding cfDNA sample. In patients with $NPM1^{mut}$ AML MRD, quantification via qPCR analysis of peripheral blood was considered equivalent to bone marrow cytology as remission control, therefore bone marrow analysis was conducted less frequently/only when relapse was suspected.

** Indication of the chimerism tests closest in time to the cfDNA sampling. A "-" indicates that no more recent chimerism analysis was conducted prior to the corresponding cfDNA sample.

*** Interval in days between the collection of bone marrow samples or chimerism analysis and the corresponding cfDNA sample.

Supplementary Table S2: Covered regions in the VariantPlex Core AML panel (10 genes).

Gene	Reference transcript	Covered exons
ASXL1	NM_015338	11-13
CEBPA	NM_004364	1
DNMT3A	NM_022552	2, 3, 5-23
DNMT3A	NM_153759	1, 2
DNMT3A	NM_175630	4
FLT3	NM_004119	8-17,19-21
IDH1	NM_005896	3, 4
IDH2	NM_002168	4, 6
JAK2	NM_004972	12-16
KIT	NM_000222	2, 8-18
NPM1	NM_002520	11
RUNX1	NM_001754	2, 3, 5-9
RUNX1	NM_001122607	1, 5

Supplementary Table S3: Covered regions in the VariantPlex Core Myeloid Panel (37 genes).

Gene	Reference transcript	Covered exons
ABL1	NM_005157	4-9, 19
ANKRD26	NM_014915	1 (c.-113-c.-134)
ASXL1	NM_015338	11-13
BCOR	NM_017745	2-7, 9-15
BCOR	NM_001123385	8
BRAF	NM_004333	11, 15
CALR	NM_004343	8, 9
CBL	NM_005188	8, 9
CEBPA	NM_004364	1
CSF3R	NM_000760	10, 14-16
CSF3R	NM_156039	17
CSF3R	NM_172313	18
DDX41	NM_016222	1-17
DNMT3A	NM_022552	2, 3, 5-23
DNMT3A	NM_153759	1, 2
DNMT3A	NM_175630	4
ETNK1	NM_018638	3
ETV6	NM_001987	1-8
EZH2	NM_004456	2-20
FLT3	NM_004119	8-21
GATA1	NM_002049	2
GATA2	NM_032638	2-6
IDH1	NM_005896	3, 4
IDH2	NM_002168	4, 6
JAK2	NM_004972	12-16
KIT	NM_000222	2, 8-18
KRAS	NM_004985	2-4
MPL	NM_005373	10,1 2
NPM1	NM_002520	11
NRAS	NM_002524	2-4
PHF6	NM_032458	9, 10
PHF6	NM_032335	2-8
PTPN11	NM_002834	3, 4, 7, 8, 12, 13
PTPN11	NM_080601	11
RUNX1	NM_001754	2, 3, 5-9
RUNX1	NM_001122607	1,5
SETBP1	NM_015559	4 (p.799-p.950)
SF3B1	NM_012433	13-18

SRSF2	NM_003016	1,2
STAG2	NM_006603	2-33
STAG2	NM_001042749	32
TET2	NM_001127208	4-11
TET2	NM_017628	3
TP53	NM_000546	1-11
TP53	NM_001276696	10
TP53	NM_001276695	10
U2AF1	NM_006758	2, 5, 6
WT1	NM_000378	1-9
WT1	NM_001198552	8
ZRSR2	NM_005089	1-11

Supplementary Table S4: Additional patient characteristics (cf. Table 1 in the main manuscript).

	N	%
AML		
primary	21	72
secondary	8	28
MDS	3	37.5
CMML	2	25.0
PV	1	12.5
Burkitt's lymphoma	1	12.5
Breast cancer	1	12.5
Number of mutations		
Median	2	
Range	1-7	
Cytogenetics*		
Normal karyotype	15	51.7
Complex karyotype	5	17.2
-7/del 7(q)	3	10.3
del 8(q)	1	3.4
del 9(q)	1	3.4
inv(10)	1	3.4
t(9;22)	1	3.4
t(9;11)	1	3.4
Trisomy 1	1	3.4
Trisomy 4	1	3.4
Trisomy 13	1	3.4
Trisomy 19	1	3.4
Trisomy 21	2	6.9
t(X;16)	1	3.4
t(2;12)	1	3.4
Allogeneic stem cell transplantation	25	86
AML	24	96
CMML, AML as relapse	1	4
CR before aHSCT	21	84
CR after aHSCT	25	100
Progression	17	59
Previous CR	13	76
Progression free survival after CR (days)		
Median	249	
Range	15-3037	
Deaths	16	55
AML	13	81
other causes	3	19

* Some patients exhibited more than one chromosomal alteration.

Supplementary Table S5: Mutant genes at initial diagnosis in 29 AML patients as reported by the reference laboratory.

Gene	Number of patients with at least one mutation per gene; n [%]
ASXL1	4 [13.8]
BCOR	1 [3.4]
CEBPA.	3 [10.3]
DNMT3A	8 [27.6]
ETV6	1 [3.4]
FLT3**	4 [13.8]
IDH1	4 [13.8]
IDH2	7 [24.1]
JAK2	1 [3.4]
NPM1	11 [37.9]
NRAS	4 [13.8]
RUNX1	4 [13.8]
SETBP1	2 [6.9]
SRSF2	3 [10.3]
TET2	5 [17.2]
TP53	4 [13.8]

Supplementary Table S6: Sequencing depth and detectable VAF in 75 cfDNA samples from 29 AML patients.

Gene	Mutation (DNA)	Mutation (Protein)	Mean depth / SD	Min./ Max.	Lowest VAF (calculated)	Lowest VAF (observed)	N (samples)
**ASXL1**	c.1934dup	p.Gly646Trpfs Ter12	1226 / 192.3	1045 / 1428	0.07	2.68	3
**ASXL1**	c.2077C>T	p.Arg693Ter	2530	2530	0.04	0.12	1
**ASXL1**	c.2194_2202del insGGGCA	p.Leu732Glyfs Ter11	778.7 / 501.8	267 / 1270	0.08	13.64	2
**BCOR**	c.2512C>T	p.Pro838Ser	195.5 / 132.2	66 / 253	0.40	99.21	2
**CEBPA**	c.71_74dup	p.Glu25Aspfs Ter27	703.5 / 248.2	405 / 969	0.10	0.1	3
**CEBPA**	c.173dup	p.His59Alafs Ter84	980	980	0.10	0.2	1
**CEBPA**	c.238_241dup	p.Leu81Argfs Ter28	691	691	0.14	n.d.	0
**CEBPA**	c.310G>T	p.Asp104Tyr	479.5 / 333	244 / 715	0.14	49.18	2
**CEBPA**	c.823G>A	p.Lys275Ter	875	875	0.11	n.d.	0
**DNMT3A**	c.670G>A	p.Ala224Thr	184.5 / 108.2	108 / 261	0.38	10.19	2
**DNMT3A**	c.1127_1147dup	p.Tyr376_382dup	433	433	0.23	0.23	1
**DNMT3A**	c.1235G>C	p.Gly412Ala	638	638	0.16	n.d.	0
**DNMT3A**	c.1457C>G	p.Ser486Cys	1044 / 304.5	713 / 1317	0.08	0.78	4
**DNMT3A**	c.1903C>T	p.Arg635Trp	845	845	0.12	n.d.	0
**DNMT3A**	c.2189G>A	p.Arg730His	426.5 / 407.5	60 / 1348	0.07	0.09	7
**DNMT3A**	c.2189G>T	p.Arg730Leu	845	845	0.12	n.d.	0
**ETV6**	c.1058G>C	p.Arg353Pro	288	288	0.35	1.04	1
**FLT3**	c.1770_1793dup	p.Tyr597_Glu598 insAspTyrValAsp PheArgGluTyr	1781 / 1896	440 / 3121	0.03	37.27	2
**FLT3**	c.2505T>G	p.Asp835Glu	114	114	0.88	26.32	1
**IDH1**	c.394C>T	p.Arg132Cys	1695 / 1540	328 / 3363	0.03	0.91	5
**IDH1**	c.395G>A	p.Arg132His	884.8 / 1008	41 / 2303	0.04	0.16	3
**IDH1**	c.395G>A	p.Arg132Leu	784.5 / 202.9	641 / 928	0.11	n.d.	0
**IDH2**	c.263G>A	p.Arg88Gln	418.2 / 226.2	99 / 850	0.12	0.3	10
**IDH2**	c.359G>A	p.Arg120Lys	916.5 / 914	115 / 2505	0.04	4.7	4
**JAK2**	c.1849G>T	p.Val617Phe	730.8 / 180.9	580 / 985	0.10	6.57	4
**NPM1**	c.860_863dup	p.Trp288Cysfs Ter12	397.8 / 193.8	148 / 918	0.11	0.16	7
**NPM1**	c.863_864 insCATG	p.Trp288Gysfs Ter12	571.7 / 219.1	355 / 843	0.12	0.48	1
**NPM1**	c.863_864 insCCTG	p.Trp288Gysfs Ter12	581.3 / 163	383 / 736	0.14	0.26	3
**NRAS**	c.34G>T	p.Gly12Cys	530	530	0.19	n.d.	0
**NRAS**	c.35G>A	p.Gly12Asp	1012 / 427.2	532 / 1590	0.06	0.11	5
**NRAS**	c.38G>A	p.Gly13Asp	534	534	0.19	n.d.	0
**NRAS**	c.182A>G	p.Gln61Arg	116	116	0.86	n.d.	0
**RUNX1**	c.457_458del insCCTC	p.Phe153Profs Ter32	1066 / 429.1	680 / 1528	0.07	0.38	1
**RUNX1**	c.511G>A	p.Asp171Asn	1829 / 1806	808 / 5034	0.02	2.93	5
**RUNX1**	c.762C>G	p.Tyr254Ter	664.6 / 333.6	84 / 910	0.11	0.48	5
**RUNX1**	c.1015_1022del	p.Ile339Valfs Ter231	621	621	0.16	n.d.	0
**SETBP1**	c.2602G>A	p.Asp868Asn	3224 / 2758	1273 / 5174	0.02	0.08	2
**SETBP1**	c.2602G>T	p.Asp868Tyr	4510	4510	0.02	3.3	1
**SETBP1**	c.2608G>A	p.Gly870Ser	4497	4497	0.02	1.27	1
**SRSF2**	c.284C>T	p.Pro95Leu	61	61	1.64	11.48	1
**SRSF2**	c.284C>G	p.Pro95Arg	126	126	0.79	n.d.	0
**TET2**	c.2604T>G	p.Phe868Leu	1273	1273	0.08	4.35	1
**TET2**	c.2737C>T	p.Gln913Ter	533.5 / 211.4	384 / 683	0.15	78.04	1
**TET2**	c.3359T>A	p.Leu1120Ter	1035 / 656.9	570 / 1499	0.07	0.13	2
**TET2**	c.3641G>A	p.Arg1214Gln	167	167	0.6	n.d.	0
**TET2**	c.4106C>A	p.Ser1369Ter	679	679	0.15	n.d.	0
**TET2**	c.5253_5254del	p.Tyr1751	204	204	0.49	n.d.	0
**TP53**	c.422G>A	p.Cys141Tyr	284 / 100.4	213 / 355	0.28	n.d.	0
**TP53**	c.742C>T	p.Arg248Trp	420	420	0.24	20.48	1
**TP53**	c.816_817insGG ACCG	p.Val272_Arg273 insGlyPro	1604	1604	0.06	n.d.	0
**TP53**	c.970del	p.Asp324Metfs Ter21	609	609	0.16	0.66	1
**Total**			776 / 849.8	41 / 5174	0.02		

SD = standard deviation; n.d. = not detectable. * formula used: x = 1 / maximum read count at mutation site ** number of samples in which this mutation could be detected

Supplementary Table S7: Sequencing depth and MRD detection in cfDNA samples.

Panel/ Sequencer	Total Readnumber > recommended					
	N	Mean Read# / SD	N (CR)	N (non-CR)	N (positive, CR)	N (positive, non-CR)
Core AML/ MiSeq	8	1,249,785 / 271,381	5	3	3	3
Core Myeloid/ MiSeq	22	4,144,453 / 779,261	20	2	16	2
Core Myeloid/ NextSeq	18	3,569,009 / 287,703	12	6	7	6
**Total**	48		37	11	**26**	**11**

Panel/ Sequencer	Total Readnumber < recommended*					
	N	Mean Read# / SD	N (CR)	N (non-CR)	N (positive, CR)	N (positive, non-CR)
Core AML/ MiSeq	0					
Core Myeloid/ MiSeq	11	2,338,915 / 715,318	9	2	3	2
Core Myeloid/ NextSeq	16	2,326,517 / 468,418	9	7	3	7
**Total**	27		18	9	**6**	**9**

*Recommended sequencing depth: Core AML 750.000 reads, Core Myeloid 3.000.000 reads

Supplementary Figure S1: MRD-status in cfDNA in AML samples sequenced at high depth.
N=37 for CR and n=11 for non-CR samples. P=0.0478 (Fisher's exact test)

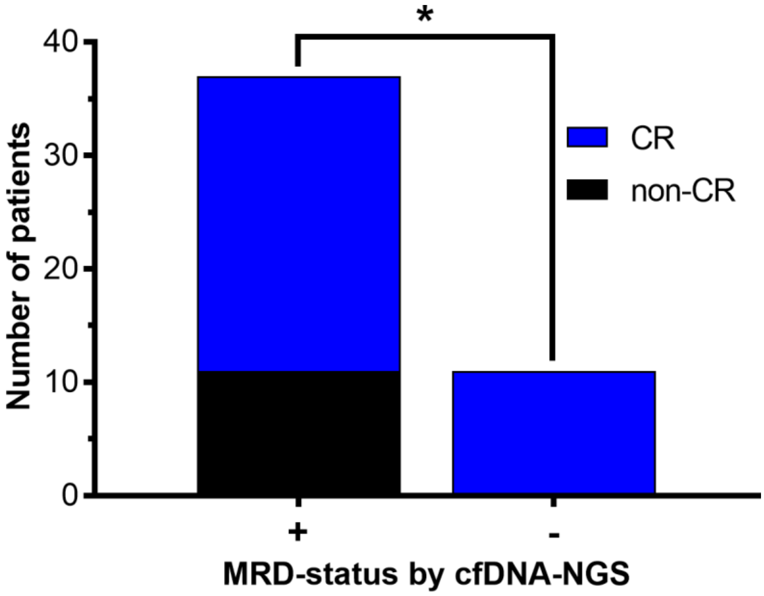

Supplementary Table S8: Mutations in cfDNA samples 29 AML patients in CR and non-CR

Gen	Mutation (DNA)	Mutation (Protein)	Number of patients	Number of samples	cfDNA positive (CR)*	cfDNA negative (CR)*	cfDNA positive (non-CR)*	cfDNA negative (non-CR)*
**ASXL1****	c.1934dup	p.Gly646Trpfs Ter12	2	3	3	0	0	0
**ASXL1**	c.2077C>T	p.Arg693Ter	1	1	1	0	0	0
**ASXL1**	c.2194_2202delins GGGCA	p.Leu732Glyfs Ter11	1	3	0	1	2	0
**BCOR**	c.2512C>T	p.Pro838Ser	1	2	0	0	2	0
**CEBPA**	c.71_74dup	p.Glu25Aspfs Ter27	1	5	1	2	2	0
**CEBPA**	c.173dup	p.His59AlafsTer84	1	1	1	0	0	0
**CEBPA**	c.238_241 dup	p.Leu81Argfs*28	1	1	0	1	0	0
**CEBPA**	c.310G>T	p.Asp104Tyr	1	2	0	0	2	0
**CEBPA**	c.823G>A	p.Lys275*	1	1	0	1	0	0
**DNMT3A**	c.670G>A	p.Ala224Thr	1	2	0	0	2	0
**DNMT3A**	c.1127_ 1147dup	p.Tyr376_382dup	1	1	1	0	0	0
**DNMT3A**	c.1235G>C	p.Gly412Ala	1	1	0	1	0	0
**DNMT3A**	c.1913C>G	p.Ser638Cys	1	4	4	0	0	0
**DNMT3A**	c.1903C>T	p.Arg635Trp	1	1	0	1	0	0
**DNMT3A**	c.2645G>A	p.Arg882His	3	9	5	2	2	0
**DNMT3A**	c.2645G>T	p.Arg882Leu	1	1	0	1	0	0
**ETV6**	c.1058G>C	p.Arg353Pro	1	1	1	0	0	0
**FLT3**	c.1770_ 1793dup	p.Tyr597_Glu598 insAspTyrValAsp PheArgGluTyr	1	6	0	4	2	0
**FLT3**	c.2505T>G	p.Asp835Glu	1	1	0	0	1	0
**IDH1**	c.394C>T	p.Arg132Cys	3	5	1	0	4	0
**IDH1**	c.395G>A	p.Arg132His	1	6	1	3	2	0
**IDH1**	c.395G>T	p.Arg132Leu	1	2	0	2	0	0
**IDH2**	c.419G>A	p.Arg140Gln	4	16	8	5	3	0
**IDH2**	c.359G>A	p.Arg120Lys	3	6	0	2	4	0
**JAK2**	c.1849G>T	p.Val617Phe	1	4	2	0	2	0
**NPM1 (Type A)**	c.860_863 dup	p.Trp288Cysfs Ter12	9	29	3	22	4	0
**NPM1 (Type B)**	c.863_864 insCATG	p.Trp288Cysfs Ter12	1	6	1	5	0	0
**NPM1 (Type D)**	c.863_864 insCCTG	p.Trp288Cysfs Ter12	1	4	3	1	0	0
**NRAS**	c.34G>A	p.Gly12Cys	1	1	0	1	0	0
**NRAS**	c.35G>A	p.Gly12Asp	2	8	5	3	0	0
**NRAS**	c.38G>A	p.Gly13Asp	1	1	0	1	0	0
**NRAS**	c.182A>G	p.Gln61Arg	1	1	0	1	0	0
**RUNX1**	c.457_458 delinsCCTC	p.Phe153Profs Ter32	1	3	1	2	0	0
**RUNX1**	c.511G>A	p.Asp171Asn	2	5	2	0	3	0
**RUNX1**	c.762C>G	p.Tyr254Ter	1	5	2	0	3	0
**RUNX1**	c.1015_ 1022del	p.Ile339Valfs Ter231	1	1	0	1	0	0
**SETBP1**	c.2602G>A	p.Asp868Asn	1	1	1	0	0	0
**SETBP1**	c.2602G>A	p.Asp868Tyr	1	1	0	0	1	0
**SETBP1**	c.2608G>A	p.Gly870Ser	1	1	0	0	1	0
**SRSF2**	c.284C>T	p.Pro95Leu	1	1	0	0	1	0
**SRSF2**	c.284C>G	p.Pro95Arg	1	1	0	1	0	0
**TET2**	c.2604T>G	p.Phe868Leu	1	1	1	0	0	0
**TET2**	c.2737C>T	p.Gln913Ter	1	2	1	1	0	0
**TET2**	c.3359T>A	p.Leu1120Ter	1	3	2	1	0	0
**TET2**	c.3641G>A	p.Arg1214Gln	1	1	0	1	0	0
**TET2**	c.4106C>A	p.Ser1369Ter	1	1	0	1	0	0
**TET2**	c.5253_ 5254del	p.Tyr1751	1	1	0	1	0	0
**TP53**	c.422G>A	p.Cys141Tyr	1	4	0	4	0	0
**TP53**	c.742C>T	p.Arg248Trp	1	1	0	0	1	0
**TP53**	c.816_817 insGGACC G	p.Val272_Arg273 insGlyPro	1	1	0	1	0	0
**TP53**	c.970del	p.Asp324Metfs Ter21	1	1	1	0	0	0

*Only samples from patients in whom the mutation was identified at initial diagnosis were included.
**The ASXL1 c.1934dup mutation is technically challenging to distinguish from an artifact at low allele frequencies, as it lies in a homopolymer region (GGGGGGGG), which promotes sequencing errors due to DNA polymerase "slippage." Therefore, this variant is unsuitable as an MRD marker.

Supplementary Table S9: NPM1-mutations as determined by NGS of cfDNA in CR- und non-CR-samples from AML patients. Samples from both BM (n=10) and PB (n=22) are included.

	**CR**	**Non-CR**	**Total**
**NPM1-mutation positive (cfDNA)**	9	3	12
**NPM1-mutation negative (cfDNA)**	20	0	20
**Total**	29	3	32

Supplementary Table S10: Chimerism vs. cfDNA analysis to detect residual AML in patients after aHSCT. Evidence for residual disease: cfDNA positive, Chimerism <90%, No evidence of disease: cfDNA negative, Chimerism ≥90%. Kappa = 0.218 (95%-CI 0.042-0.394).

	**cfDNA positive**	**cfDNA negative**	**Total**
**Chimerism <90%**	6	0	6
**Chimerism ≥90%**	16	13	29
**Total**	22	13	35

Supplementary Figure S2: Distribution of donor chimerism in CR-samples that were MRD positive as determined by cfDNA analysis. n=21, the horizontal line indicates the median.

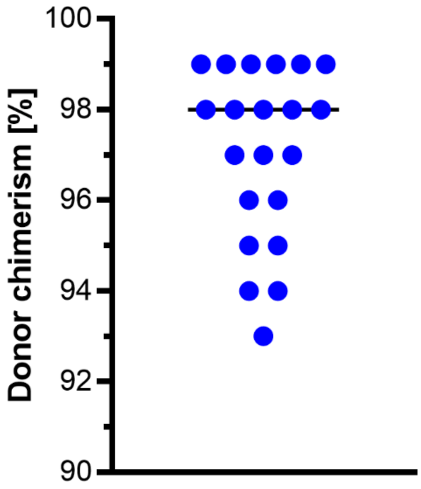

Supplementary Table S11: Mutations in AML patients at initial diagnosis and at relapse. Mutations that had been reported by the reference laboratory at initial diagnosis (from BM analyses) are indicated in bold. VAF refers to the cfDNA sample at relapse. Mutations detected at initial diagnosis but not present at relapse are not listed.

Patient	Gene	c.DNA	Protein	VAF [%]
5	***RUNX1***	**c.762C>G**	**p.Tyr254Ter**	**13.74**
	IDH2	**c.419G>A**	**p.Arg140Gln**	**12.19**
	***FLT3*-ITD**			**n.d.**
	DNMT3A	c.2645G>A	p.Arg882His	13.69
	BCOR	c.3487C>T	p.Arg1163Ter	16.11
	WT1	c.831C>A	p.Cys277Ter	3.2
11	***JAK2***	**c.1849G>T**	**p.Val617Phe**	**6.57**
	RUNX1	**c.511G>A**	**p.Asp171Asn**	**3.22**
	DNMT3A	c.2645G>A	p.Arg882His	8.01
	TET2	c.5396dup	p.Met1800AspfsTer6	4.92
15	***TP53***	**c.472C>T**	**p.Arg248Trp**	**20.48**
	GATA2	c.925_929dup	p.Gly312AlafsTer16	16.75
16	***IDH1***	**c.394C>T**	**p.Arg132Cys**	**8.46**
	SRSF2	**c.284C>T**	**p.Pro95Leu**	**11.48**
	BRAF	c.1803A>T	p.Lys601Asn	5.33
	TET2	c.2827C>T	p.Gln943Ter	6.72
17	***DNMT3A***	**c.1913C>G**	**p.Ser638Cys**	**29.84**
	IDH2	**c.419G>A**	**p.Arg140Gln**	**31.06**
	NPM1	**c.863_864insCCTG**	**p.Trp288CysfsTer12**	**22.83**
	*NRAS **	c.38G>A	p.Gly13Asp	0.08
	CEBPA	c.59del	p.Leu20ArgfsTer21	6.99
21	***RUNX1***	**c.511G>A**	**p.Asp171Asn**	**62.77**
	*IDH1 ***	c.394C>T	p.Arg132Cys	47.85
	SETBP1	c.2602G>A	p.Asp868Asn	45.05
	ASXL1	c.2061del	p.Cys687Ter	48.81
	CSF3R	c.2221C>T	p.Gln741Ter	46.03
25	***NPM1***	**c.860_863dup**	**p.Trp288CysfsTer12**	**0.16**
	CBL	c.1248T>G	p.Cys416Trp	3.31
28	***DNMT3A***	**c.2189G>A**	**p.Arg730His**	**46.67**
	FLT3	**c.1770_1793**	**p.Tyr597_Glu598insAspTyr ValAspPheArgGluTyr**	**37.27**
	IDH1	**c.395G>A**	**p.Arg132His**	**68.25**
	NPM1	**c.860_863dup**	**p.Trp288CysfsTer12**	**42.06**
	CBL	c.1380_1382del	p.Asp460del	2.73

VAF = variant allele frequency, n.d. = not detectable
*: This variant was detected in a bone marrow sample during relapse (reference laboratory) after cfDNA sampling. **: This variant was detected in an external laboratory during therapy.
